# Reliability and predictive validity of two scales of self-rated health in China: results from China Health and Retirement Longitudinal Study (CHARLS)

**DOI:** 10.1186/s12889-022-14218-1

**Published:** 2022-10-05

**Authors:** Yuwei Pan, Jitka Pikhartova, Martin Bobak, Hynek Pikhart

**Affiliations:** Research Department of Epidemiology and Public Health, University College London, 1-19 Torrington Place, WC1E 6BT London, UK

**Keywords:** Reliability, Validity, Health status indicators, China, Longitudinal studies

## Abstract

**Background:**

Despite the widespread use of the single item self-rated health (SRH) question, its reliability has never been evaluated in Chinese population.

**Methods:**

We used data from the China Health and Retirement Longitudinal Study, waves 1–4 (2011–2019). In wave 1, the same SRH question was asked twice, separated by other questions, on a subset of 4533 subjects, allowing us to examine the test–retest reliability of SRH. In addition, two versions of SRH questions (the WHO and US versions) were asked (*n* = 11,429). Kappa (κ), weighted kappa (${\kappa}_{w}$), and polychoric correlation coefficient (ρ) were used for reliability assessment. Cox proportional-hazards models were estimated to assess the predictive validity of SRH measurement for mortality over 7 years of follow up. To do so, relative index of inequality (RII) and slope index of inequality (SII) were estimated for each SRH scale.

**Results:**

There was moderate to substantial test–retest reliability (κ = 0.54, ${\kappa}_{w}$=0.63) of SRH; 31% of respondents who used the same scale twice changed their ratings after answering other questions. There was strong positive association between the two SRH measured by the two scales (*ρ* > 0.8). Compared with excellent/very good SRH, adjusted hazard ratios (HR) of death are 2.30 (95% CI, 1.70–3.13) for the US version and 1.86 (95% CI, 1.33–2.60) for the WHO version. Using slope indices of inequality, the WHO version estimated slightly larger mortality differences (RII = 3.50, SII = 15.53) than the US version (RII = 3.25, SII = 14.80).

**Conclusions:**

In Chinese middle-aged and older population, the reliability of SRH is generally good, although the two commonly used versions of SRH scales could not be compared directly. Both indices predict mortality, with similar predictive validity.

**Supplementary Information:**

The online version contains supplementary material available at 10.1186/s12889-022-14218-1.

## Background

The single item self-rated health (SRH) has been widely seen as an indicator of overall health status. SRH has been shown to be an independent predictor of morbidity and mortality [1–7]. There are several explanations about the association between the negative evaluation of one’s health and mortality. Two of them are that negative evaluation reflects awareness of underlying disease burden, and negative evaluation reflects a weak sense of mastery [8, 9].

SRH is usually measured by asking individuals to evaluate their health on a five-point scale (could be more or less categories) with or without a given reference point (self-comparative or age-comparative) [10, 11]. The five-point scale of SRH without reference to self or age might be a better predictor of mortality than self-comparative and age-comparative SRH, and more appropriate for longitudinal research [12, 13]. There are two commonly used versions of five-point scale of SRH. The scale recommended by WHO-Europe uses categories “very good, good, fair, bad, very bad” [14], while the other version (mainly used in the US) used categories “excellent, very good, good, fair, poor”. However, although being mixed used in China, it remains unclear whether the two versions are equivalent among Chinese population.

Moreover, previous studies have shown that the predictive validity of mortality of SRH may differ between populations and certain subgroups [15, 16], and poor SRH (“poor” or less than “good”) was a stronger predictor of morbidity and mortality, compared with good SRH [17, 18].

The validity of SRH refers to the accuracy of the measure, while the reliability of SRH refers to the consistency and stability of the measure. The evidence on the reliability of SRH among adults is limited. We found only 4 studies on reliability of SRH in adults [19–22], and all of them were conducted in Western populations. Although SRH has been widely used as a predictor of morbidity and mortality in China, its reliability has never been assessed. In addition, current findings on the reliability of SRH between age subgroups are inconsistent. A Swedish study reported good overall reliability of SRH, and the reliability is better among older men compared with younger men (*P* < 0.01), but not among women [20]. However, in a study from Australia, kappa scores of SRH reliability were lower among older age groups, although weighted kappa indicates no such difference [21]. Furthermore, factors indicating socioeconomic status (SES) including education, occupation, and income were found to be related to the reliability and predictive ability of SRH [21, 22]. A study conducted among US adults reported lower reliability of SRH among ethnic minorities and people with lower education [22].

To our knowledge, none of previous studies compared the two commonly used versions of five-point scale of SRH in Asian population, and none of the previous studies evaluated the reliability of SRH scales in Chinese population. To fill those gaps, the current study compared the two versions of SRH and assessed the reliability of SRH among nationally representative sample of Chinese residents. In addition, the current study also assessed the predictive validity of mortality of the two SRH scales among Chinese middle-aged and older population.

## Methods

### Study population

This study used data from China Health and Retirement Longitudinal Study (CHARLS) [23], which is a nationally representative survey of Chinese residents aged 45 years or over along with their spouses. It covers information on family, health status and functioning, healthcare and insurance, work circumstances (work, retirement and pension), and economic status of community residents [23]. The national baseline survey (wave 1) was conducted between 2011 and 2012. Totalling 17,708 respondents were involved [24]. Response rate for the baseline survey is 80.5% [24]. Follow-up surveys were conducted every two years and the latest national wave (wave 4) was conducted between 2018 and 2019 [25]. In the current study, CHARLS wave 1 was used for the SRH reliability assessment and waves 1 to 4 were used for the predictive validity (of mortality) assessment.

### Design of the self-rated health measurement

Two versions of the five-point scale of SRH were used to measure general health status in CHARLS wave 1 (2011), wave 2 (2013), and wave 3 (2015). In the face-to-face interview, respondents were asked with questions “Would you say your health is excellent, very good, good, fair, or poor?”(The US version, scale 1) and “Would you say your health is very good, good, fair, poor, or very poor?”(WHO version, scale 2). Every respondent was asked to rate their health status twice, once at the beginning of the Health Status and Functioning Section and again at the end of that section (separated by questions on disease history, lifestyle, and health behaviours). Order of the two questions was randomly assigned. However, the design of the SRH measurement in CHARLS wave 1 is special.

Among 17,708 CHARLS wave1 respondents, 15,962 individuals rated their general health status using both or one of the two SRH scales at the beginning and the end of the Health Status and Functioning Section. We divided the respondents into three groups according to their responses to the two SRH scales. Group 1 used scale 1 at the beginning of the Health Status and Functioning Section and scale 2 at the end of that section. Group 2 used scale 2 at the beginning of the Health Status and Functioning Section and scale 1 at the end. Group 3 used scale 2 twice, once at the beginning of the Health Status and Functioning Section and again at the end. This special design provided an opportunity to study the reliability of SRH in terms of (1) the test–retest reliability of the same SRH scale measured by scale 2; (2) the effect of different SRH scale versions; (3) the effect of SRH question orders; (4) and the effect of other health-related questions between two SRH measurements.

### Analytical sample

**Fig. 1:**
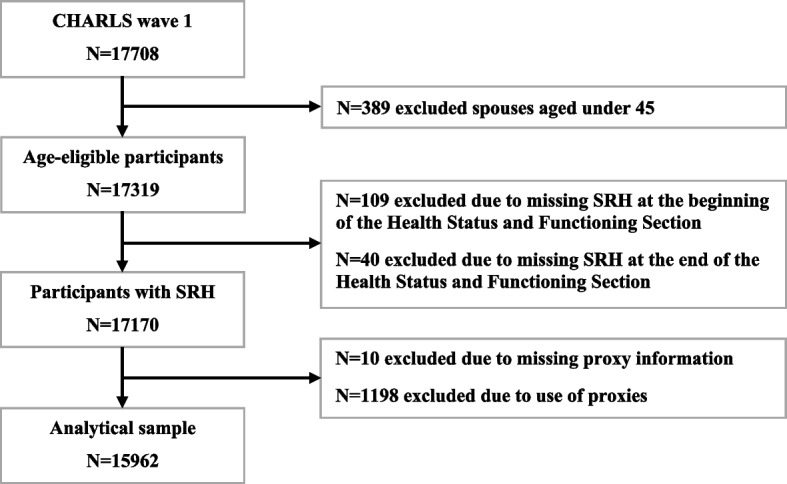
Flowchart of the sample selection procedure for self-rated health reliability and predictive validity assessment

**Fig. 2:**
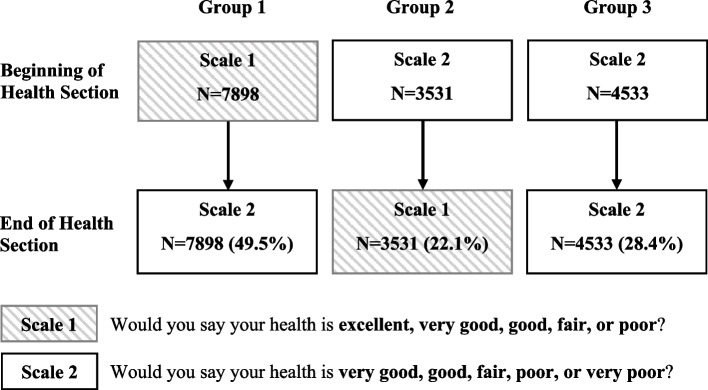
Answering frequency of self-rated health questions in the analytical sample

The analytical sample was defined as respondents aged 45 years or older, including both main respondents and age-eligible spouses, who reported SRH both at the beginning and the end of the Health Status and Functioning Section without the use of any proxy. Sample selection procedure was shown in Fig. 1. Totalling 15,962 CHARLS wave 1 respondents (9,301 main respondents and 6,661 age-eligible spouses) were included in the analytical sample. Answering frequency of SRH questions in the analytical sample was shown in Fig. 2.

### Statistical methods

First, we examined the distribution of SRH responses according to the order of questions and cross-tabulated the distribution of SRH responses to scale1 and scale 2 when combined group 1 with group 2. Second, to test the reliability of SRH, we used four measurements: (1) Proportion of agreement ($a$); (2) polychoric correlation coefficient (ρ) of two ordinal variables of SRH, assuming underlying bivariate normal distribution [26]. It was estimated by maximum likelihood [27], with -1 indicating perfect negative association, 1 indicating perfect positive association, and 0 indicating statistical independence; (3) Cohen’s kappa statistic ($\kappa$) [28, 29]. Kappa statistic is a coefficient used to measure the degree of agreement, and is calculated as $\mathrm{\kappa}\text{=}\frac{{p}_{o}-{p}_{c}}{1-{p}_{c}}$, where ${p}_{o}$ is the proportion of units in which the judges agreed, and ${p}_{c}$ is the proportion of units for which agreement is expected by chance [28]; (4) and weighted kappa (${\kappa}_{w}$) [30]. Weighted kappa is defined as $\kappa_w=\frac{\sum w_{ij}p_{oij}-\sum w_{ij}p_{cij}}{w_{max}-\sum w_{ij}p_{cij}},(i,j=1\dots\mathrm k)$, where ${}{w}_{ij}$ is the agreement weight, ${p}_{oij}$ is the proportion of the joint judgement (observed cell proportion), and ${p}_{cij}$ is the proportion in the cell expected by chance [30]. Weight used in the calculation of ${\kappa}_{w}$ can be found in supplementary data table A1. For the first three measurements, we recoded SRH into three categories (“Positive” including “Excellent”, “Very good”, and “Good”; “Fair”; and “Negative” including “Poor” and “Very poor”) when comparing different scales. ${\kappa}_{w}$ was calculated based on original five-point SRH scales.

To assess the effect of SRH version, we compared responses to scale1 and scale 2 in group 1, group 2, and in combined sample (group 1 and group 2), separately. The first two comparisons also reflect the effect of question orders and the effect of other health-related questions between two SRH questions.

To assess test–retest (intra-rater) reliability, we compared the SRH measured at the beginning and at the end of the Health Status and Functioning Section among group 3 respondents. For this comparison, we used three-category SRH and five-point scale SRH, separately. In addition, we assessed the test–retest (intra-rater) reliability of SRH (based on original five-point scale 2) according to sample characteristics including age, sex, area type, education, chronic disease history, and major accidental injuries.

To assess the predictive validity of mortality of SRH, Cox proportional-hazards models were estimated, and hazard rate ratio (HR) and 95% confidence interval (CI) were calculated. The associations between SRH measured by scale 1 and scale 2 with all-cause mortality were assessed among group 1 and group 2 respondents. Proportional hazards assumption was tested based on Schoenfeld residuals. Covariates including age, sex, area type, education, chronic diseases, and major accidental injuries were added to the model consecutively. The few respondents (*n* = 190) with missing data in covariates were analysed as a separate category. Interaction terms between SRH and each independent variable were used to identify potential effect modification. Likelihood ratio test (LRT) was used to assess whether the model fit was improved. Six regression models were presented in the results.

Regression-based relative index of inequality (RII) and slope index of inequality (SII) by two five-point SRH scales were estimated to measure the magnitude of inequalities in mortality rate (MR). We estimated RII (mortality rate ratio) to indicate relative inequality and SII (mortality rate difference) to indicate absolute inequality. RII was estimated with age- and sex- adjusted Poisson regression model. It was the ratio of the mortality of people with the worst SRH (x = 1) to the best SRH (x = 0). SII was calculated by the following formula: $$SII=2*MR*(RII-1)/(RII+1)$$ [31]. Large scores on RII and SII implies large differences in mortality rate between the better and worse SRH conditions [32].

CHARLS adopted a stratified multi-stage probabilities proportional to size (PPS) random sampling strategy [24]. To account for this complex survey design, we adjusted for baseline individual weights in the analysis. SRH information and sample characteristics were drawn from wave 1. Mortality data was from CHARLS waves 2 to 4. All the analyses were performed using Stata/MP 16.1 [33].

## Results

Table 1 shows the distribution of SRH responses according to the order of questions (percentages may not total 100% due to rounding). Generally, compared with the first inquiry, health was better when asked at the end, with proportions of positive/neutral answers (excellent, very good, good, and fair) increased, and proportions of negative answers (poor and very poor) decreased.

**Table 1:** Distribution of self-rated health responses according to the order of questions

**Scale 1 categories**	At beginning (*n* = 7898)	At the end (*n* = 3531)	**Scale 2 categories**	At beginning (*n* = 8064)	At the end (*n* = 12,431)
Excellent	0.7%	1.1%	-	-	-
Very good	8.3%	9.9%	Very good	5.8%	6.1%
Good	16.2%	21.7%	Good	16.1%	17.3%
Fair	46.6%	48.7%	Fair	47.0%	51.2%
Poor	28.2%	18.6%	Poor	25.9%	21.1%
-	-	-	Very poor	5.2%	4.4%

Distribution of responses to scale 2 is more balanced than that of scale 1. Proportions of “very good” and “very poor” are similar in scale 2, while there are 27.5% and 17.5% differences between “excellent” and “poor” options in scale 1. “Fair” category took the largest proportion. Nearly half of the respondents chose “fair” on all four occasions. In general, health (in terms of the meaning of the word) measured by scale 1 is better than it measured by scale 2. On the second inquiry, 32.7% of respondents chose positive answers and 18.6% chose negative answers when using scale 1, while only 23.4% of respondents chose positive answers and 25.5% chose negative answers when using scale 2. The stratified results of Table 1 by groups can be found in supplementary data table A2 and results adjusted for baseline weights is in table A3.

Table 2 shows the cross-tabulation of scale 1 and scale 2 responses when combined both directions (directions: scale 1 – scale 2 and scale 2 – scale 1, among group 1 and group 2 respondents). Answers were concordant mainly according to the meaning of the category instead of its relative position. Overall, 65.2% (*n* = 7,448) of respondents choose categories with the same meaning and 19.3% (*n* = 3,981) choose the one in the same position. Results adjusted for baseline weights can be found in supplementary data table A4.

**Table 2:** Cross-tabulation of self-rated health measured by scale 1 and scale 2 (*n* = 11,429)

Scale 1	Scale 2						
	Excellent	Very good	Good	Fair	Poor	Very poor	Total
Excellent	-	66	21	9	0	0	96
Very good	-	414	387	185	19	1	1006
Good	-	143	1096	719	82	6	2046
Fair	-	105	460	4194	597	47	5403
Poor	-	10	65	617	1744	442	2878
Very poor	-	-	-	-	-	-	-
Total	-	738	2029	5724	2442	496	11,429

Table 3 shows the cross-tabulation of scale 2 responses at the beginning and the end of the Health Status and Functioning Section (among group 3 respondents). 68.9% (*n* = 3,125) of respondents chose the same category and 31.1% (*n* = 1,408) changed their ratings after answering questions on disease history, lifestyle, and health behaviours. Results adjusted for baseline weights can be found in supplementary data table A5. Cross-tabulation on other occasions can be found in supplementary data (table A6, table A7, table A8, table A9, table A10, table A11, and table A12).

**Table 3:** Cross-tabulation of self-rated health measured at two occasions (*n* = 4533)

Scale 2 (Beginning of the Health Section)	Scale 2 (End of the Health Section)					
	Very good	Good	Fair	Poor	Very poor	Total
Very good	137	56	41	2	0	236
Good	60	407	197	14	6	684
Fair	47	232	1674	139	12	2104
Poor	5	35	381	773	69	1263
Very poor	2	3	28	79	134	246
Total	251	733	2321	1007	221	4533

Reliability statistics was presented in Table 4. When both scales were used to measure SRH in the same population, proportions of agreement ($a$) are higher when scale 1 was used before scale 2 ($a$ =75.7%, $a$ =74.4% versus $a$ =71.6%). kappa ($\kappa$) values are also higher when scale 1 was used first, with $\kappa$ of 0.62 indicating substantial agreement and $\kappa$ of 0.55 and 0.60 indicating moderate agreement. In addition, polychoric correlation coefficients (ρ) over 0.8 indicate a strong positive association between the two SRH variables measured by different scales (ρ of inter-scale comparisons based on five-point scales are 0.81, 0.76, and 0.79).

**Table 4:** Reliability of self-rated health, China Health and Retirement Longitudinal Study 2011

Comparisons	Num. of pairs	Agreement (%)	Polychoric correlation	Kappa	Weighted Kappa
Inter-scale					
Group1-Scale1 vs. Group1-Scale2	7898	75.7	0.83	0.62	-
Group2-Scale2 vs. Group2-Scale1	3531	71.6	0.81	0.55	-
Scale 1 vs. Scale 2	11,429	74.4	0.81	0.60	-
Intra-scale					
Group3-Scale2: begin vs. end	4533	74.8	0.82	0.60	-
Group3-Scale2: begin vs. end (Five-point scale)	4533	68.9	0.79	0.54	0.63

In terms of the test–retest reliability of SRH (intrarater/intra-scale), agreement is higher when SRH was categorized into three categories ($a$=74.8% versus $a$=68.9%). $\kappa$ of 0.60 and 0.54 indicate moderate agreement. In the comparison based on the original five-point scale, weighted kappa (${\kappa}_{w}$) of 0.63 indicates substantial agreement. Furthermore, ρ of 0.82 and 0.79 indicate a strong positive association between the SRH measured at the beginning and the end. Results adjusted for baseline weights can be found in supplementary data table A13.

Table 5 shows the test–retest reliability statistics of SRH (based on five-point scale 2) according to sample characteristics (for the first column, percentages may not total 100% due to rounding). Generally, although the agreement level is slightly higher for age group 45–54, there is no linear relationship between age and SRH agreement. $\kappa$ of 0.52–0.55 and ${\kappa}_{w}$ of 0.61–0.64 indicate moderate and substantial agreement, respectively. Moreover, agreement level is higher in the urban area, and among people with higher education level. Whether diagnosed with chronic diseases does not distinguish the agreement levels. However, respondents who experienced major accidental injuries such as traffic accidents, have lower level of agreement. Overall, there is moderate to substantial test–retest reliability of SRH among CHARLS respondents. Results adjusted for baseline weights can be found in supplementary data table A14.

**Table 5:** Test–retest reliability of self-rated health according to sample characteristics (*n* = 4533)

	**N, %**	**Agreement (%)**	**Polychoric correlation**	**Kappa**	**Weighted kappa**
Age					
45–54	1608 (35.5)	70.5	0.80	0.55	0.64
55–64	1683 (37.1)	68.0	0.79	0.52	0.61
65 or over	1242 (27.4)	68.1	0.78	0.53	0.62
Sex					
Male	1223 (27.0)	69.5	0.80	0.55	0.63
Female	3309 (73.0)	68.9	0.79	0.53	0.62
Missing	1	-	-	-	-
Area type					
Rural	2778 (61.3)	66.6	0.77	0.51	0.60
Urban	1755 (38.7)	72.7	0.82	0.58	0.66
Education					
Illiterate	1551 (34.2)	66.9	0.75	0.51	0.60
Lower than elementary school	822 (18.1)	68.5	0.79	0.54	0.63
Elementary school	859 (19.0)	68.0	0.79	0.51	0.60
Middle school	864 (19.1)	71.4	0.84	0.55	0.65
High school or above	435 (9.6)	74.2	0.86	0.60	0.68
Missing	2	-	-	-	-
Chronic diseases					
Yes	3061 (67.5)	68.7	0.76	0.53	0.60
No	1431 (31.6)	69.3	0.79	0.52	0.61
Missing	41 (0.9)	-	-	-	-
Major accidental injuries					
Yes	372 (8.2)	64.5	0.73	0.48	0.56
No	4136 (91.2)	69.5	0.80	0.54	0.63
Missing	25 (0.6)	-	-	-	-
Total	4533 (100)	68.9	0.79	0.54	0.63

Table 6 shows the results from Cox regression. Of 11,429 respondents who measured SRH using both scale 1 and scale 2 at baseline (wave 1), 47 people died in the year of baseline survey (2011), 2,550 lost to follow up (censored) and 967 died during 7 years of follow-up. Totalling 72,531 person-years of observation. “Excellent” and “Very good” in scale 1 were combined due to the small number of respondents in “Excellent”. And “Poor” and “Very poor” in scale 2 were combined. Proportional hazards assumption was tested using Schoenfeld residuals. *P* = 0.15 (SRH measured by scale 1) and 0.42 (SRH measured by scale 2) in the global test indicated the assumption was met.

**Table 6:** Hazard rate ratio of death (HR), mortality rates per 1,000 person-years (MR), relative index of inequality (RII) and slope index of inequality (SII) among Chinese middle-aged and older adults (*n* = 11,429)

Self-rated health		N, %	Model 1		Model 2		Model 3		MR per 1,000 person-years	RII (95% CI)	SII (95% CI)
			**HR (95% CI)**	***P* value**	**HR (95% CI)**	***P* value**	**HR (95% CI)**	***P* value**			
**Scale 1**	Excellent/Very good	1102 (9.64)	Ref		Ref		Ref		13.98	3.25 (2.58,4.09)	14.80 (12.34,16.97)
	Good	2046 (17.90)	1.25 (0.90,1.74)	0.18	1.26 (0.90,1.75)	0.18	1.24 (0.89,1.73)	0.20			
	Fair	5403 (47.27)	1.45 (1.08,1.95)	0.01	1.45 (1.07,1.95)	0.02	1.39 (1.03,1.88)	0.03			
	Poor	2878 (25.18)	2.59 (1.92,3.49)	< 0.001	2.46 (1.82,3.31)	< 0.001	2.30 (1.70,3.13)	< 0.001			
**Scale 2**	Very good	738 (6.46)	Ref		Ref		Ref		13.98	3.50 (2.76,4.43)	15.53 (13.09,17.66)
	Good	2029 (17.75)	0.91 (0.63,1.31)	0.62	0.90 (0.63,1.30)	0.58	0.90 (0.62,1.29)	0.56			
	Fair	5724 (50.08)	1.10 (0.79,1.52)	0.56	1.08 (0.78,1.50)	0.65	1.04 (0.75,1.45)	0.81			
	Poor/Very poor	2938 (25.71)	2.11 (1.53,2.93)	< 0.001	1.98 (1.43,2.75)	< 0.001	1.86 (1.33,2.60)	< 0.001			

Model 1: adjusted for age and sex

Model 2: additionally adjusted for area type and education

Model 3: additionally adjusted for chronic diseases and major accidental injuries

*CI* confidence interval, *HR* hazard rate ratio, *MR* mortality rate, *RII* relative index of inequality, *SII* slope index of inequality

The association between SRH measured by scale 1 and all-cause mortality is shown in Table 6. In model 1 which was adjusted for age and sex, compared with the reference group (“Excellent” and “Very good”), those with fair and poor SRH had 1.45 (95% CI, 1.08–1.95) and 2.59 (95% CI, 1.92–3.49) times the hazard of death, respectively. In model 2, hazard ratio of poor SRH reduced to 2.46 (95% CI, 1.82–3.31) after additionally adjusted for area type and education. And in model 3, after additional adjustment of chronic diseases and major accidental injuries, HRs of respondents with fair and poor SRH reduced to 1.39 (95% CI, 1.03–1.88) and 2.30 (95% CI, 1.70–3.13), respectively.

Association between SRH measured by scale 2 and mortality is also shown in Table 6. In the age and sex adjusted model, compared with the reference group “Very good”, fair and poor/very poor SRH are associated with 1.10 (95% CI, 0.79–1.52) times and 2.11 (95% CI, 1.53–2.93) times the hazard of death, respectively. After additional adjustment for area type and education in model 2, HR of poor/very poor SRH reduced to 1.98 (95% CI, 1.43–2.75). In model 3, HR of respondents with poor/very poor SRH reduced to 1.86 (95% CI, 1.33–2.60) after additionally adjusted for chronic diseases and major accidental injuries.

For both SRH measured by scale 1 and scale 2, age is an important confounder in the association between SRH and all-cause mortality. The association attenuated substantially after adjusting for age (attenuation > 10%). However, additional adjustment of sex increased the effect of this association. There is weak evidence for interactions between education and SRH measured by the two scales (*P* = 0.08 and 0.06, respectively), however, according to the results of LRT, including the interaction terms improve the prediction (LRT: *P* = 0.03 and 0.003, respectively). Results adjusted for baseline weights can be found in supplementary data table A15. Weighted results stratified by education can be found in supplementary data table A16. Moreover, compared with same categories in scale 1, “Fair” and “Poor” categories in scale 2 is less predictive of death, and “Very poor” is highly predictive of death.

## Discussion

### Summary of key findings

To our knowledge, this is the first study to investigate the reliability of two commonly used versions of SRH measurement in China, using nationally representative data from CHARLS.

Generally, there was moderate to substantial test–retest reliability of SRH in Chinese middle-aged and older adults. 31.1% of respondents who used scale 2 twice changed their ratings after answering questions on disease history, lifestyle, and health behaviours. When both scales were used for the same individuals, the reliability is higher when question order is scale 1 – scale 2 instead of scale 2 – scale 1. In addition, there was no linear relationship between age and SRH reliability. However, the reliability was higher in urban area, among people with higher education level, and lower among people who experienced major accidental injuries.

Both SRH versions were significantly associated with all-cause mortality among Chinese middle-aged and older adults, with age acting as an important confounder of the association between SRH and mortality.

Moreover, there was strong positive association between SRH measured by the two commonly used versions, indicating both scales measured the same latent variable. Furthermore, responses to the two different scales were concordant mainly according to the meaning of the category instead of its relative position. However, although measuring the same construct, there were still differences between SRH responses to the two scales, general health condition measured by scale 1 is better than it measured by scale 2. In addition, scale 2 reflects greater mortality inequalities in both relative and absolute terms than scale 1.

### Findings in the context of existing studies

Findings of the current study indicate moderate to substantial reliability of SRH among Chinese middle-aged and older population. Among group 3 respondents who used scale 2 twice, 31.1% of them changed their ratings after answering a set of health-related questions. This result is similar to previous findings from Australia, in which 28% of respondents changed their ratings after answering a set of health-related questions [21]. And consistent with findings from the US [22], we found there is no linear relationship between age and SRH reliability, with the middle-aged group (the US study: 40–59 years; current study: 45–54 years) had the highest agreement level, and education being linearly related to SRH reliability. Study from Swedish population reported excellent reliability among older men aged 46–75 (${\kappa}_{w}$=0.82) and lower reliability among older women of the same age (${\kappa}_{w}$=0.58) [20]. The study used three-category SRH. However, we did not find such sex difference, neither with three-category SRH nor original five-category SRH. The study from Canada was conducted among 18 women with mean age of 68 [19], and did not compare the consistency of test–retest results, therefore we could not compare our results with it.

The study on SRH and mortality from Hong Kong used three-category age-comparative SRH and self-comparative SRH to measure health status among respondents aged 65 or over living in Elderly Health Centres in Hong Kong [11]. It reported that compared with better age-comparative SRH, worse age-comparative SRH was positively associated with all-cause mortality (fully adjusted model: HR = 1.24, 95% CI, 1.17–1.31), while worse self-comparative SRH did not (fully adjusted model: HR = 0.91, 95% CI, 0.86–0.96). Compared with that study, the present study is more representative of the Chinese population, as we used nationally representative sample from household residents instead of institutions, and covered younger population aged 45–64. Our results indicate a positive association between fair/poor/very poor SRH and all-cause mortality among Chinese residents aged 45 or over. And this result is in the same direction with findings on age comparative SRH from Hong Kong.

Jürges and colleagues compared the WHO version (scale 2) and US version (scale 1) of SRH using data from the Survey of Health, Ageing and Retirement in Europe (SHARE) [34]. Consistent with the findings in European population, health condition measured by scale 1 is better than it measured by scale 2; and there is higher levels of literal concordance (verbally consistent) than relative concordance (consistent in terms of position). However, there are also differences. Contrary to the findings from European population, in the current study among Chinese population, scale 1 has more skewed distribution and scale 2 has more balanced distribution. Moreover, European population are more likely to choose better SRH. In the present study, about half of the respondents chose “fair” in both scales, and around 1% of respondents chose “excellent” in scale 2 (Table 1). However, in the European population, less than 30% of respondents chose “fair” in both scales, 7.5% chose “excellent” in the US version, and 67.1% and 60.5% chose excellent/very good and very good/good, respectively [34]. This is in accordance with the finding that Chinese older adults were more likely to report worse SRH compared with their American counterparts [35], which might be related to the traditional pursuit of moderation in China. In addition, results of current study indicate higher reliability of SRH among urban population and people with higher education level. This may be due to the fact that these population groups are more health conscious, but this is only speculation, and this finding may need further exploration.

Another study using data from the English Longitudinal Study of Ageing (ELSA) assessed the effect of question order and response-choices of SRH [36]. Consistent with their findings from older population living in England, we found that among Chinese older residents, SRH measured after the health-related section gives better general health status, compared with that measured before the health section.

### Limitations

There are several limitations of this study. First, the reliability assessment was based on cross-sectional data. Therefore, we could not take account of the effect of longer time period. Second, the predictive validity of SRH was assessed only in terms of all-cause mortality, which, although being representative, could not show the potential differences between specific health outcomes. Third, as the SRH information was only from baseline wave, we could not assess the change in the predictive validity of SRH overtime. However, one previous study reported that the predictive validity of mortality of SRH is increasing over time [37].

### Implications

Our study suggests measurement error in SRH among Chinese middle-aged and older adults. Responses to SRH questions depend on the version of the scale, question order, sample characteristics such as age, education, area type, and whether experienced major accidental injuries. Researchers should consider the effect of the above factors when designing studies or interpreting their results. Both SRH versions predict all-cause mortality among Chinese middle-aged and older adults, however, given the difference in categories between the two scales, the effect estimates appear to differ, although the RII and SII were similar. In addition, the WHO version and US version SRH are both effective in measuring health status, but they could not be compared directly. When using SRH to measure health status, Chinese population tend to report worse health than Western population, therefore, the results need to be interpreted cautiously.

## Conclusions

This study for the first time assessed the reliability of SRH among Chinese population. Overall, there was moderate to substantial test–retest reliability of SRH in Chinese middle-aged and older adults, and the reliability was higher in some subgroups. Both SRH versions can predict mortality among Chinese middle-aged and older adults, with similar predictive validity. Although the two SRH versions measured the same latent variable, they could not be compared directly among Chinese middle-aged and older population.

## Supplementary Information

**Additional file 1: Table A1.**  Weight used in the calculation of weighted kappa. **Table A2.**  Distribution of self-rated health responses according to the order of questions stratified by groups. **Table A3.  **Distribution of self-rated health responses according to the order of questions after adjusting for baseline weights. **Table A4. **Cross-tabulation of self-rated health measured by scale 1 and scale 2 after adjusting for baseline weights. **Table A5.  **Cross-tabulation of self-rated health measured at two occasions after adjusting for baseline weights. **Table A6.**  Cross-tabulation of self-rated health responses at two occasions among group 1 and group 2 respondents (*n*=11429). **Table A7.**  Cross-tabulation of three-point scale 1 and scale 2 responses among group 1 respondents (*n*=7898). **Table A8.**  Cross-tabulation of three-point scale 1 and scale 2 responses among group 2 respondents (*n*=3531). **Table A9.**  Cross-tabulation of three-point scale 1 and scale 2 responses among group 1 and group 2 respondents (*n*=11429). **Table A10.**  Cross tabulation of three-point scale 2 responses among group  3 respondents (*n*=4533). **Table A11.**  Distribution of self-rated health responses among group 1 respondents (*n*=7898). **Table A12.  **Distribution of self-rated health responses among group 2 respondents (*n*=3531). **Table A13.  **Reliability of self-rated health after adjusting for baseline weights. **Table A14.** Test-retest reliability of self-rated health according to sample characteristics after adjusting for baseline weights. **Table A15. **Hazard rate ratio of death (HR), mortality rates per 1,000 person-years (MR), relative index of inequality (RII) and slope index of inequality (SII) among Chinese middle-aged and older adults (*n*=11429) (Results adjusted for baseline weights). **Table A16. **Self-rated health and age-adjusted hazard rate ratio of death (HR) stratified by education. 

## Data Availability

The datasets generated and/or analysed during the current study are available from China Health and Retirement Longitudinal Study project home page, http://charls.pku.edu.cn/en/.

## Abbreviations

SRH: Self-rated health
CHARLS: China Health and Retirement Longitudinal Study
SES: Socioeconomic status
IRB: Institutional Review Board
HR: Hazard rate ratio
CI: Confidence interval
LRT: Likelihood ratio test
RII: Relative index of inequality
SII: Slope index of inequality
MR: Mortality rate
PPS: Probabilities proportional to size
SHARE: Survey of Health, Ageing and Retirement in Europe
ELSA: English Longitudinal Study of Ageing
