# Supplementary material for: Reliability and predictive validity of two scales of self-rated health in China: results from China Health and Retirement Longitudinal Study (CHARLS)
Source: BMC Public Health. 2022 Oct 5;22:1863. doi: 10.1186/s12889-022-14218-1 (PMC9536015; doi:10.1186/s12889-022-14218-1)
Supplement: Supplementary file 1 — Additional file 1: Table A1. Weight used in the calculation of weighted kappa. Table A2. Distribution of self-rated health responses according to the order of questions stratified by groups. Table A3. Distribution of self-rated health responses according to the order of questions after adjusting for baseline weights. Table A4. Cross-tabulation of self-rated health measured by scale 1 and scale 2 after adjusting for baseline weights. Table A5. Cross-tabulation of self-rated health measured at two occasions after adjusting for baseline weights. Table A6. Cross-tabulation of self-rated health responses at two occasions among group 1 and group 2 respondents (n=11429). Table A7. Cross-tabulation of three-point scale 1 and scale 2 responses among group 1 respondents (n=7898). Table A8. Cross-tabulation of three-point scale 1 and scale 2 responses among group 2 respondents (n=3531). Table A9. Cross-tabulation of three-point scale 1 and scale 2 responses among group 1 and group 2 respondents (n=11429). Table A10. Cross tabulation of three-point scale 2 responses among group 3 respondents (n=4533). Table A11. Distribution of self-rated health responses among group 1 respondents (n=7898). Table A12. Distribution of self-rated health responses among group 2 respondents (n=3531). Table A13. Reliability of self-rated health after adjusting for baseline weights. Table A14. Test-retest reliability of self-rated health according to sample characteristics after adjusting for baseline weights. Table A15. Hazard rate ratio of death (HR), mortality rates per 1,000 person-years (MR), relative index of inequality (RII) and slope index of inequality (SII) among Chinese middle-aged and older adults (n=11429) (Results adjusted for baseline weights). Table A16. Self-rated health and age-adjusted hazard rate ratio of death (HR) stratified by education. [file 12889_2022_14218_MOESM1_ESM.docx]

**Supplementary data**

**Table A1.** Weight used in the calculation of weighted kappa

| **Weight used in the calculation of weighted kappa** | | | | |
| --- | --- | --- | --- | --- |
| 1.0000 | 0.7500 | 0.5000 | 0.2500 | 0.0000 |
| 0.7500 | 1.0000 | 0.7500 | 0.5000 | 0.2500 |
| 0.5000 | 0.7500 | 1.0000 | 0.7500 | 0.5000 |
| 0.2500 | 0.5000 | 0.7500 | 1.0000 | 0.7500 |
| 0.0000 | 0.2500 | 0.5000 | 0.7500 | 1.0000 |

**Table A2.** Distribution of self-rated health responses according to the order of questions stratified by groups

| **Scale 1 categories** | At beginning  (n=7898) | At the end  (n=3531) | **Scale 2 categories** | At beginning  (n=8064) | At the end  (n=12431) |
| --- | --- | --- | --- | --- | --- |
| **Group 1** |  |  |  |  |  |
| Excellent | 0.7% | - | - | - | - |
| Very good | 8.3% | - | Very good | - | 6.4% |
| Good | 16.2% | - | Good | - | 17.9% |
| Fair | 46.6% | - | Fair | - | 51.2% |
| Poor | 28.2% | - | Poor | - | 20.5% |
| - | - | - | Very poor | - | 4.1% |
| **Group 2** |  |  |  |  |  |
| Excellent | - | 1.1% | - | - | - |
| Very good | - | 9.9% | Very good | 6.6% | - |
| Good | - | 21.7% | Good | 17.4% | - |
| Fair | - | 48.7% | Fair | 47.7% | - |
| Poor | - | 18.6% | Poor | 23.4% | - |
| - | - | - | Very poor | 4.9% | - |
| **Group 3** |  |  |  |  |  |
| Excellent | - | - | - | - | - |
| Very good | - | - | Very good | 5.2% | 5.5% |
| Good | - | - | Good | 15.1% | 16.2% |
| Fair | - | - | Fair | 46.4% | 51.2% |
| Poor | - | - | Poor | 27.9% | 22.2% |
| - | - | - | Very poor | 5.4% | 4.9% |

**Table A3.** Distribution of self-rated health responses according to the order of questions after adjusting for baseline weights

| **Scale 1 categories** | At beginning  (n=7898) | At the end  (n=3531) | **Scale 2 categories** | At beginning  (n=8064) | At the end  (n=12431) |
| --- | --- | --- | --- | --- | --- |
| Excellent | 0.8% | 0.9% | - | - | - |
| Very good | 8.4% | 12.1% | Very good | 7.0% | 6.2% |
| Good | 17.3% | 22.3% | Good | 17.4% | 18.6% |
| Fair | 47.0% | 47.3% | Fair | 45.8% | 50.8% |
| Poor | 26.4% | 17.4% | Poor | 25.0% | 20.2% |
| - | - | - | Very poor | 4.8% | 4.2% |

**Table A4.** Cross-tabulation of self-rated health measured by scale 1 and scale 2 after adjusting for baseline weights

| **Scale 1** | **Scale 2** | | | | | | |
| --- | --- | --- | --- | --- | --- | --- | --- |
|  | Excellent | Very good | Good | Fair | Poor | Very poor | Total |
| Excellent | - | 70 | 19 | 6 | 0 | 0 | 95 |
| Very good | - | 490 | 422 | 170 | 17 | 1 | 1100 |
| Good | - | 145 | 1167 | 757 | 88 | 7 | 2164 |
| Fair | - | 93 | 481 | 4178 | 573 | 56 | 5381 |
| Poor | - | 9 | 61 | 562 | 1669 | 388 | 2689 |
| Very poor | - | - | - | - | - | - | - |
| Total | - | 807 | 2150 | 5673 | 2347 | 452 | 11429 |

**Table A5.** Cross-tabulation of self-rated health measured at two occasions after adjusting for baseline weights

| **Scale 2**  **(Beginning of the Health Section)** | **Scale 2 (End of the Health Section)** | | | | | |
| --- | --- | --- | --- | --- | --- | --- |
|  | Very good | Good | Fair | Poor | Very poor | Total |
| Very good | 151 | 65 | 44 | 2 | 0 | 262 |
| Good | 59 | 477 | 197 | 16 | 4 | 753 |
| Fair | 43 | 225 | 1634 | 148 | 11 | 2061 |
| Poor | 5 | 37 | 360 | 743 | 76 | 1221 |
| Very poor | 1 | 3 | 29 | 71 | 132 | 236 |
| Total | 259 | 807 | 2264 | 980 | 223 | 4533 |

**Table A6.** Cross-tabulation of self-rated health responses at two occasions among group 1 and group 2 respondents (n=11429).

| **Beginning of the Health Section** | **End of the Health Section** | | | | | | |
| --- | --- | --- | --- | --- | --- | --- | --- |
|  | Excellent | Very good | Good | Fair | Poor | Very poor | Total |
| Excellent | 0 | 44 | 10 | 4 | 0 | 0 | 58 |
| Very good | 22 | 414 | 305 | 138 | 10 | 0 | 889 |
| Good | 11 | 225 | 1096 | 530 | 30 | 1 | 1893 |
| Fair | 5 | 152 | 649 | 4194 | 355 | 11 | 5366 |
| Poor | 0 | 19 | 117 | 859 | 1744 | 311 | 3050 |
| Very poor | 0 | 1 | 5 | 36 | 131 | 0 | 173 |
| Total | 38 | 855 | 2182 | 5761 | 2270 | 323 | 11429 |

**Table A7.** Cross-tabulation of three-point scale 1 and scale 2 responses among group 1 respondents (n=7898).

| **Scale 1** | **Scale 2** | | | |
| --- | --- | --- | --- | --- |
|  | Positive | Fair | Negative | Total |
| Positive | 1423 | 534 | 35 | 1992 |
| Fair | 427 | 2955 | 301 | 3683 |
| Negative | 69 | 552 | 1602 | 2223 |
| Total | 1919 | 4041 | 1938 | 7898 |

**Table A8.** Cross-tabulation of three-point scale 1 and scale 2 responses among group 2 respondents (n=3531).

| **Scale 2** | **Scale 1** | | | |
| --- | --- | --- | --- | --- |
|  | Positive | Fair | Negative | Total |
| Positive | 704 | 138 | 6 | 848 |
| Fair | 379 | 1239 | 65 | 1683 |
| Negative | 73 | 343 | 584 | 1000 |
| Total | 1156 | 1720 | 655 | 3531 |

**Table A9.** Cross-tabulation of three-point scale 1 and scale 2 responses among group 1 and group 2 respondents (n=11429).

| **Scale 1** | **Scale 2** | | | |
| --- | --- | --- | --- | --- |
|  | Positive | Fair | Negative | Total |
| Positive | 2127 | 913 | 108 | 3148 |
| Fair | 565 | 4194 | 644 | 5403 |
| Negative | 75 | 617 | 2186 | 2878 |
| Total | 2767 | 5724 | 2938 | 11429 |

**Table A10.** Cross tabulation of three-point scale 2 responses among group 3 respondents (n=4533).

| **Scale 2**  **(Beginning of the Health Section)** | **Scale 2 (End of the Health Section)** | | | |
| --- | --- | --- | --- | --- |
|  | Positive | Fair | Negative | Total |
| Positive | 660 | 238 | 22 | 920 |
| Fair | 279 | 1674 | 151 | 2104 |
| Negative | 45 | 409 | 1055 | 1509 |
| Total | 984 | 2321 | 1228 | 4533 |

**Table A11.** Distribution of self-rated health responses among group 1 respondents (n=7898).

| **Scale 1**  **(Beginning of the Health Section)** | **Scale 2 (End of the Health Section)** | | | | | |
| --- | --- | --- | --- | --- | --- | --- |
|  | Very good | Good | Fair | Poor | Very poor | Total |
| Excellent | 44 | 10 | 4 | 0 | 0 | 58 |
| Very good | 283 | 258 | 106 | 8 | 0 | 655 |
| Good | 96 | 732 | 424 | 26 | 1 | 1279 |
| Fair | 73 | 354 | 2955 | 290 | 11 | 3683 |
| Poor | 8 | 61 | 552 | 1291 | 311 | 2223 |
| Total | 504 | 1415 | 4041 | 1615 | 323 | 7898 |

**Table A12.** Distribution of self-rated health responses among group 2 respondents (n=3531).

| **Scale 2**  **(Beginning of the Health Section)** | **Scale 1 (End of the Health Section)** | | | | | |
| --- | --- | --- | --- | --- | --- | --- |
|  | Excellent | Very good | Good | Fair | Poor | Total |
| Very good | 22 | 131 | 47 | 32 | 2 | 234 |
| Good | 11 | 129 | 364 | 106 | 4 | 614 |
| Fair | 5 | 79 | 295 | 1239 | 65 | 1683 |
| Poor | 0 | 11 | 56 | 307 | 453 | 827 |
| Very poor | 0 | 1 | 5 | 36 | 131 | 173 |
| Total | 38 | 351 | 767 | 1720 | 655 | 3531 |

**Table A13.** Reliability of self-rated health after adjusting for baseline weights

| **Comparisons** | **Num. of pairs** | **Agreement (%)** | **Kappa** | **Weighted Kappa** |
| --- | --- | --- | --- | --- |
| Inter-scale |  |  |  |  |
| Group1-Scale1 vs. Group1-Scale2 | 7898 | 76.2 | 0.62 | - |
| Group2-Scale2 vs. Group2-Scale1 | 3531 | 71.9 | 0.56 | - |
| Scale 1 vs. Scale 2 | 11429 | 74.8 | 0.60 | - |
| Intra-scale |  |  |  |  |
| Group3-Scale2:  begin vs. end | 4533 | 75.2 | 0.72 | - |
| Group3-Scale2:  begin vs. end  (Five-point scale) | 4533 | 69.2 | 0.55 | 0.63 |

**Table A14.** Test-retest reliability of self-rated health according to sample characteristics after adjusting for baseline weights

|  | **N, %** | **Agreement (%)** | **Kappa** |
| --- | --- | --- | --- |
| Age |  |  |  |
| 45-54 | 1564 (34.5) | 70.7 | 0.56 |
| 55-64 | 1623 (35.8) | 68.6 | 0.54 |
| 65 or over | 1346 (29.7) | 68.2 | 0.53 |
| Sex |  |  |  |
| Male | 1233 (27.2) | 68.6 | 0.54 |
| Female | 3300 (72.8) | 69.4 | 0.55 |
| Missing | - | - | - |
| Area type |  |  |  |
| Rural | 2552 (56.3) | 66.4 | 0.51 |
| Urban | 1981 (43.7) | 72.9 | 0.60 |
| Education |  |  |  |
| Illiterate | 1505 (33.2) | 66.5 | 0.51 |
| Lower than elementary school | 807 (17.8) | 70.7 | 0.57 |
| Elementary school | 852 (18.8) | 68.4 | 0.53 |
| Middle school | 879 (19.4) | 73.0 | 0.59 |
| High school or above | 485 (10.7) | 69.8 | 0.54 |
| Missing | - | - | - |
| Chronic diseases |  |  |  |
| Yes | 3033 (66.9) | 68.8 | 0.53 |
| No | 1455 (32.1) | 69.7 | 0.54 |
| Missing | 45 (1.0) | - | - |
| Major accidental injuries |  |  |  |
| Yes | 381 (8.4) | 65.8 | 0.49 |
| No | 4120 (90.9) | 69.7 | 0.55 |
| Missing | 32 (0.7) | - | - |
| Total | 4533 (100) | 69.2 | 0.55 |

**Table A15.** Hazard rate ratio of death (HR), mortality rates per 1,000 person-years (MR), relative index of inequality (RII) and slope index of inequality (SII) among Chinese middle-aged and older adults (n=11429) (Results adjusted for baseline weights)

| **Self-rated health** | | **N, %** | **Model 1** | | **Model 2** | | **Model 3** | | **MR per 1,000 person-years** | **RII**  **(95% CI)** | **SII**  **(95% CI)** |
| --- | --- | --- | --- | --- | --- | --- | --- | --- | --- | --- | --- |
|  |  |  | **HR (95% CI)** | ***P* value** | **HR (95% CI)** | ***P* value** | **HR (95% CI)** | ***P* value** |  |  |  |
| **Scale 1** | Excellent/Very good | 1200 (10.5) | Ref |  | Ref |  | Ref |  | 13.98 | 3.56  (2.54,4.98) | 15.70  (12.16,18.61) |
|  | Good | 2160 (18.9) | 1.11 (0.75,1.66) | 0.60 | 1.13 (0.76,1.67) | 0.54 | 1.14 (0.77,1.68) | 0.52 |  |  |  |
|  | Fair | 5383 (47.1) | 1.31 (0.90,1.89) | 0.16 | 1.34 (0.93,1.93) | 0.12 | 1.33 (0.93,1.92) | 0.12 |  |  |  |
|  | Poor | 2686 (23.5) | 2.57 (1.75,3.78) | <0.001 | 2.47 (1.67,3.64) | <0.001 | 2.46 (1.67,3.62) | <0.001 |  |  |  |
| **Scale 2** | Very good | 811 (7.1) | Ref |  | Ref |  | Ref |  | 13.98 | 3.74  (2.75,5.09) | 16.16  (13.05,18.78) |
|  | Good | 2149 (18.8) | 0.72 (0.63,1.31) | 0.14 | 0.72 (0.47,1.11) | 0.14 | 0.73 (0.47,1.12) | 0.15 |  |  |  |
|  | Fair | 5680 (49.7) | 0.93 (0.79,1.52) | 0.72 | 0.93 (0.62,1.40) | 0.74 | 0.94 (0.63,1.40) | 0.76 |  |  |  |
|  | Poor/Very poor | 2789 (24.4) | 1.91 (1.53,2.93) | 0.002 | 1.81 (1.19,2.76) | 0.006 | 1.82 (1.19,2.76) | 0.005 |  |  |  |
| Model 1: adjusted for age and sex.  Model 2: additionally adjusted for area type and education.  Model 3: additionally adjusted for chronic diseases and major accidental injuries.  CI, confidence interval; HR, hazard rate ratio; MR, mortality rate; RII, relative index of inequality; SII, slope index of inequality. | | | | | | | | | | | |

**Table A16.** Self-rated health and age-adjusted hazard rate ratio of death (HR) stratified by education

|  |  | **Age-adjusted HR (95% CI)** | | | | |
| --- | --- | --- | --- | --- | --- | --- |
|  |  | Illiterate  n=2842 | Lower than elementary school  n= 2,047 | Elementary school  n= 2,568 | Middle school  n= 2,390 | High school or above  n= 1,575 |
| **Scale 1** | Excellent/Very good | Ref | Ref | Ref | Ref | Ref |
|  | Good | 1.37 | 0.59 | 6.53  (1.39,30.71) | 0.99  (0.42,2.33) | 0.54 |
|  | Fair | 1.76 | 0.68 | 6.23  (1.36,28.48) | 1.53  (0.76,3.07) | 0.48 |
|  | Poor | 2.19 | 1.26 | 12.28  (2.70,55.78) | 4.12  (1.65,10.31) | 2.08 |
| **Scale 2** | Very good | Ref | Ref | Ref | Ref | Ref |
|  | Good | 1.35 | 0.78 | 1.18  (0.45,3.10) | 0.56  (0.22,1.44) | 0.02 |
|  | Fair | 1.19 | 0.96 | 1.42  (0.58,3.49) | 1.36  (0.60,3.11) | 0.31 |
|  | Poor/  Very poor | 2.04 | 1.67 | 2.72  (1.11,6.68) | 3.25  (1.22,8.64) | 0.85 |
